# Rethinking self-identification in neurodivergent communities: barriers, harms, and the need for change

**DOI:** 10.3389/frcha.2025.1682129

**Published:** 2025-10-13

**Authors:** Alice Newton, Aidan Flinn, James Downs, Laura Richmond

**Affiliations:** ^1^ Manchester University NHS Foundation Trust, Manchester, United Kingdom; ^2^ Division of Psychology and Mental Health, School of Health Sciences, Faculty of Biological, Medical and Health Sciences, Manchester Academic Health Science, University of Manchester, Manchester, United Kingdom; ^3^ Independent Researcher, Cardiff, United Kingdom; ^4^ Department of Clinical, Education and Health Psychology, University College London, London, United Kingdom

**Keywords:** autism, ADHD, neurodivergence, self-diagnosis, self-identity

## Abstract

Self-identification of neurodivergence is increasingly common, yet remains contentious in psychiatric, medical, and public discourse. While concerns have been raised about the reliability and potential impact on clinical services, these discussions often neglect the systemic barriers and personal experiences that can lead to self-identification in the first place. This article explores why individuals might self-identify, highlighting inequities in diagnostic access and clinician biases, as well as individual experiences and beliefs around clinical diagnosis. We argue that while self-identification can be a personal preference, it is often a survival strategy in the face of inaccessible, exclusionary, and sometimes harmful diagnostic systems. Drawing on theories of epistemic justice, we critique medical gatekeeping that delegitimises self-identification and propose a shift towards neurodiversity-affirming care. Rather than policing self-identification, we suggest that efforts should be made to address structural failures that render it necessary. Until access to clinical diagnosis become equitable, self-identification will remain an essential and legitimate means of understanding neurodivergence.

## 1 Introduction

**Figure 1:**
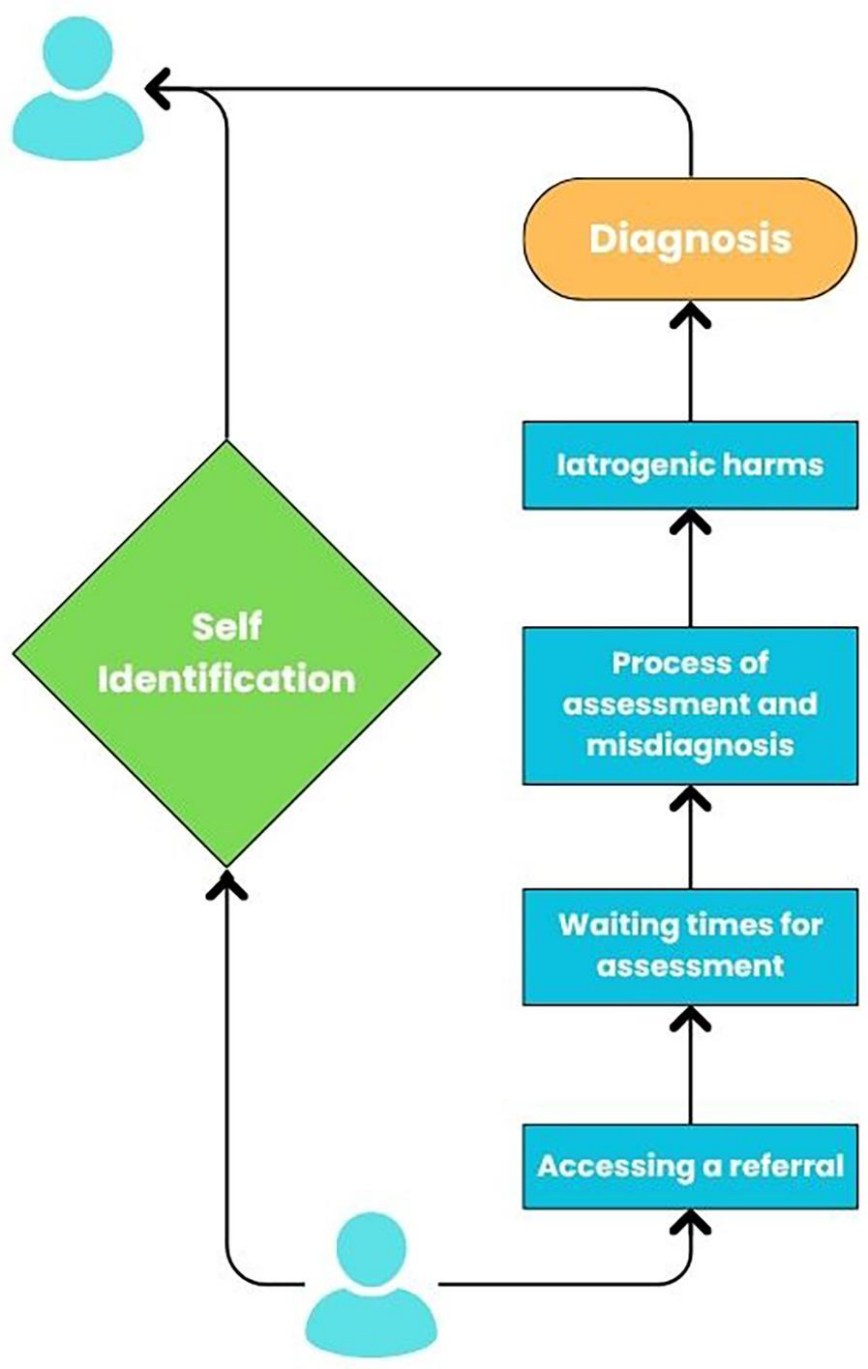
Routes to understanding and support for neurodivergent individuals.

Conversations around neurodivergence and self-diagnosis are increasingly prevalent, as more people begin to identify as neurodivergent without—or prior to—clinical diagnosis. This shift has sparked debate within psychiatric, medical, and public discourse, with concerns raised about the validity of self-diagnosis, its implications for clinical services, and its potential impact on how neurodivergence is understood and supported (1). Discussions around self-diagnosis can often overlook the lived experiences of those who identify and are diagnosed as neurodivergent, as well as the barriers and systemic factors that lead to self-identification. Self-identification of neurodivergence is a legitimate expression of an identity shaped through a social rather than a medical lens and is an essential part of understanding the way an individual may perceive and experience the world. This has been shown to improve self-understanding and self-acceptance (2–5). As such, in this article, we explore the barriers and personal viewpoints that make self-identification essential for so many neurodivergent individuals, the harms perpetuated by exclusionary diagnostic processes, and the importance of shifting towards more inclusive, neuro-affirmative models of care (see Figure 1).

We write from the perspective of neurodivergent individuals, both self-identified and formally diagnosed. We prefer the term “self-identification” over “self-diagnosis” because it reflects a recognition of neurodivergence that does not necessarily rely on a medical model. Many of us do not see neurodivergence as an illness or deficit, and self-identification can be a meaningful, legitimate, and necessary means of understanding ourselves and the world. While our identities are by no means heterogenous, we acknowledge that as white British people who have been able to access higher education, we carry privileges which may obscure experiences of other neurodivergent individuals. Nonetheless, we offer our own views and experiences to balance the points made by others in the field related to self-identification, we highlight significant problems in the provision of timely, appropriate healthcare, and we present an alternative point of view based on our lived experiences. The views, language and terminology expressed in this paper are appropriate at the time of writing, but this may change as our understanding advances.

## 2 Background

### 2.1 Barriers to clinical diagnosis

#### 2.1.1 Obtaining a referral to a specialist service

In the UK, the first step to pursuing a clinical diagnosis of neurodivergence, such as Autism or ADHD, is to see your general practitioner [GP; (6)]. This often begins with scheduling and attending some form of appointment: even at this very early stage there may already be multiple barriers present for certain populations. Doherty et al. (7) found that 80% of autistic adults in their study had difficulty attending GP appointments, which drops to 37% for the non-autistic adults. Some of the reasons provided by autistic adults around why they found visiting the GP difficult were: not feeling understood, difficulty communicating with their doctor and the waiting room environment (7).

When it comes to attending an appointment and exploring a referral to specialist services, success can depend on the education, approach and confidence of the GP or clinician. One study found that 39.4% of GP's reported having received no formal training around autism and many GPs reported struggling with their confidence in their ability to identify and provide support to autistic people (6). People exploring whether they are autistic have reported concerns around their GP's knowledge and understanding of autism, which is often a source of stress during the process (8). Other research has found similar outcomes when it comes to ADHD, with both service users and GP's reporting that whilst GP's had some knowledge of ADHD, most GPs had limited understanding and obtaining a referral to specialist services usually relied on the individual already having a strong idea that they might have ADHD (9). This highlights an important issue: even if a clinical diagnosis is sought out and given, some element of self-identification can predate it.

Another potential barrier to obtaining a referral to a specialist service is clinician bias. As the clinician acts as a gatekeeper to specialist services, a referral relies on their perceptions and opinion. For example, research as highlighted significant racial biases in the referral and assessment process, leading to misidentification and exclusion of people from ethnic minority communities (10, 37). Despite this, GPs have consistently expressed their desire to learn and provide a better service for neurodivergent individuals, further highlighting the need for further understanding and training opportunities (6, 9).

#### 2.1.2 Diagnostic assessments

NHS diagnostic services are severely under-resourced, with waiting times to see specialist teams varying dramatically depending on location. Data from the UK shows that a person seeking an ADHD assessment might wait 12 weeks in one part of the country and over 10 years in another (11). By the time an individual joins a waiting list, they may have already spent years questioning their experiences and seeking answers. Faced with yet another long wait, many understandably turn to self-identification to make sense of their identity, find communities, and access the support they need.

Another difficulty within the diagnostic process can be a failure to account for adaptive strategies neurodivergent individuals use to “pass” as neurotypical. Recent research has highlighted that masking could be a key reason why many neurodivergent people do not receive a clinical diagnosis, particularly in childhood (12). Masking, which is also sometimes termed social camouflaging, is the act of trying to disguise and compensate for neurodivergent traits in an effort to cope in a predominantly neurotypical social world (13, 14). It is important to note that masking often comes at a great cost to the individual. Masking can increase psychological distress and exhaustion (15, 16). The process of learning not to mask is often a long and difficult one fraught with lots of emotions (17), it might be difficult for someone to immediately unmask, or even identify what constitutes their mask, for an appointment with someone they have never met before.

Clinician bias can also affect the outcome of a diagnostic assessment. Research has shown that children from minoritised ethnic backgrounds are less likely than white children to receive an ADHD diagnosis (18–20) and are more likely to be misdiagnosed with “conduct disorders” instead (21). Women and people of marginalised genders face similar barriers, with outdated assumptions about “typical” presentations leading to frequent misdiagnosis with personality disorders or other mental health conditions (16, 22). These biases create a self-perpetuating cycle in which certain groups are underdiagnosed, reinforcing a narrow and exclusionary view of neurodivergence. These concerns deter people from seeking out a clinical diagnosis alongside as making the process of assessment more challenging for both the assessor and the person being assessed.

#### 2.1.3 Choosing not to pursue clinical diagnosis

There are people who choose not to pursue clinical diagnosis due to the fear of possible social, personal, or professional repercussions. Concerns can range from losing opportunities such as the ability to emigrate, to being treated differently by peers (23, 24). These concerns have been validated by recent research with Stagg and Belcher (25), reporting that adults who received a clinical diagnosis of autism in later life reported difficult consequences, including one participant losing a job due to discrimination. Thus, it is understandable that some people may be deterred from seeking a clinical diagnosis.

There are a proportion of people who may reject engaging with clinical care due to difficult past experiences and fear of mistreatment/harm from clinical services. Aves (26) critiques how distress in neurodivergent individuals can be misinterpreted by clinicians. For example, autistic distress responses—which may involve shutdowns, intense emotional reactions, or withdrawal—are often mislabelled as “manipulative” or “attention-seeking”, rather than understood as valid expressions of overwhelm. Brede et al. (27) documented cases where autistic women were dismissed by clinicians, with some explicitly told that they were “too articulate” or “too socially competent” to be autistic. This misinterpretation could result in inappropriate interventions or diagnoses, including coercive treatments and invalidating responses. Similarly, autistic distress can be misread as symptoms of mental illness, resulting in inappropriate use of medication, misdiagnosis, and even psychiatric hospitalisation, shown in multiple case studies (28, 29). These experiences can lead to deep mistrust of healthcare providers and avoidance of services, with neurodivergent people relying instead on self-identification and community support. In this context, self-identification is not an act of defiance but one of self-preservation.

Another important factor in the decision not to pursue a clinical diagnosis can be the ways in which the medical model can pathologize neurodivergence (24). The medical model often uses deficit-based language, for example, categorising traits as impairments rather than valid ways of being. This can alienate individuals who see their neurodivergence as an identity rather than a disorder. As a result, some people may reject clinical diagnosis in favour of self-identification, as this may offer a more accurate and affirming understanding of their experiences.

## 3 Discussion

Self-identification can have a myriad of benefits for neurodivergent people. Many neurodivergent people have spent years, sometimes decades, feeling different but unable to explain why (25). Neurodivergent people report that self-identification can lead to a greater understanding one's experiences, challenges, and strengths, often leading to increased self-compassion and acceptance (24, 30). We believe that every neurodivergent person has a right to these benefits, whether they can access a clinical diagnosis or not.

One perceived difficulty with self-identification is that it may limit the types of support someone can access for example, medication for ADHD or access to post-diagnostic support. However, at present, post-diagnostic support varies enormously between services, and some teams do not provide any post-diagnostic support. Subsequently, it has been suggested that clinical diagnosis can represent a false hope for getting support and solutions (24). While a clinical diagnosis may increase a person's access to support and accommodations, it is not legally required for accommodations to be made, and these can be at the discretion of an employer (31, 32). There are also types of support that can be accessed without a clinical diagnosis, such as online communities and some peer support groups.

Some critics argue that the increase in people self-identifying neurodivergence risks ‘diluting’ neurodivergent diagnoses (1). However, we posit that this perspective fails to acknowledge the rigid gatekeeping that prevents many from obtaining a diagnosis in the first place. Neurodivergent people are not responsible for ensuring that clinicians take them seriously—clinicians should take all patients seriously, regardless of whether they gain their understanding of themselves through self-identification or clinical assessment.

Another criticism of self-identification often presented is the concern that self-identification is inaccurate, in part due to the lack of training the individual has had compared with a professional (1, 33). No matter whether a person receives a clinical diagnosis or self-identifies, there will always be times where it may be judged by either themselves or someone else as inaccurate. However, this could happen with any physical or mental health condition. Diagnostic errors occur in medical settings as well (34). When it comes to neurodivergence, however, this is used as an argument against self-identification. It is worth considering such views might be due to attitudes towards neurodivergence in general, not just self-identification.

## 4 Conclusion

The numerous barriers to obtaining a clinical diagnosis mean that self-identification can be the only available pathway for neurodivergent individuals, especially those from minoritised backgrounds. When formal systems fail to recognise or understand neurodivergence, individuals are left to seek alternative means of understanding themselves—whether through online communities, self-reflection, or peer-led initiatives. Despite well-documented barriers to clinical diagnosis, those who self-identify are frequently met with scepticism and dismissal. Addressing these biases requires a fundamental shift in how neurodivergence is understood and assessed, moving away from deficit-based models that reinforce existing inequalities and towards neurodiversity-affirming frameworks that recognise the validity of lived experience and acknowledge people's ability to be the judge of their own experiences.

At the heart of debates about self-identification is often a question of power: who gets to define neurodivergence, and whose knowledge is considered legitimate? Historically, clinical expertise has been privileged over lived experience, with medical gatekeeping determining who is recognised as neurodivergent. Self-identification is often dismissed because it challenges traditional models of clinical authority. However, this dismissal is itself a form of epistemic injustice—a concept describing how certain groups are systematically disbelieved or denied credibility. Rather than treating self-identification as a “lesser” form of knowledge, it should be viewed as an essential component of a neurodiversity-affirming model of care.

Reforming diagnostic systems is necessary, but it is not enough. A truly inclusive model of neurodivergence must move beyond deficit-based frameworks toward approaches that value neurodivergent ways of being as valid expressions of humanity. The “double empathy problem” (38) highlights that the communication breakdown between neurodivergent and neurotypical people is mutual, not a deficit within neurodivergent individuals. This challenges traditional diagnostic approaches that assume neurodivergent communication styles are inherently “impaired” rather than differently structured. Neurodiversity-affirming care models, such as those proposed by Monique Botha, advocate for moving away from rigid diagnostic criteria and toward individualised, strengths-based approaches. These models align with co-production principles, which have been increasingly emphasised in policy—but rarely implemented effectively. Whilst the inclusion of neurodivergent voices is already mandated in mental health research (35) and service design (36), there is little evidence that these commitments have led to meaningful change.

The rise in self-identification among neurodivergent individuals is not a crisis—it is a response to crisis. Instead of viewing self-identification as a threat, we should recognise it as a response to systemic failures and an opportunity for change. Diagnosis remains an essential tool for many, but it is not the only valid means of understanding neurodivergence. Given the barriers, biases, and harms embedded in current diagnostic frameworks, self-identification can be an act of self-preservation and of epistemic resistance. Rather than policing self-identification, the focus should be on addressing the systemic barriers that render it necessary. A system that truly values neurodivergent perspectives must move beyond gatekeeping and deficit models toward approaches that centre self-knowledge, lived experience, and community validation. Until formal diagnostic systems are reformed to be equitable, affirming, and accessible, self-identification will remain a crucial—and entirely legitimate—pathway for recognising neurodivergence.

## 5 Clinical recommendations

We posit a range of clinical recommendations for practitioners who wish to incorporate self-identification into their practice. These are both attitudinal and practical, recognising the role that affirming and coproduced approaches play in creating more effective and equitable care:

• Adopt a neurodivergent-affirming stance: Approach self-identification as a valid and valuable expression of lived experience, rather than as a deficit or barrier to care. Actively welcome and encourage conversations about self-identification as part of a person-centred approach. (or similar!)
• Embed coproduction: Ensure that a diverse range of neurodivergent people have a meaningful role in designing, delivering, and evaluating the services they need and that lived experiences shape assessment pathways and clinical practice.
• Provide neurodivergent-led training to equip healthcare professionals (including referrers) with knowledge and confidence to support self-identifying neurodivergent individuals.
• Embed self-identification in referral and assessment: Ensure referrals and assessments capture the individual's own understandings and experiences of self-identification, alongside clinical perceptions. The diagnostic process should be undertaken in partnership with service users, not done to them or on them.
• Support community connection: Develop social prescribing, peer networks, caregiver support, and links with neurodivergent groups across diverse communities.

## Data Availability

The original contributions presented in the study are included in the article/Supplementary Material, further inquiries can be directed to the corresponding author.
